# The Difference

**Published:** 1859-06

**Authors:** 


### THE DIFFERENCE.

When a simpleton wants to get well, he buys something "to take," a philosopher gets something "to do;" and it is owing to the circumstance, that the latter has been in a minority almost undistinguishable in all nations and ages, that doctors are princes, instead of paupers; live like gentlemen, instead of cracking rocks for the turnpike.

